# On-chip integration of metasurface-doublet for optical phased array with enhanced beam steering

**DOI:** 10.1515/nanoph-2022-0697

**Published:** 2023-01-16

**Authors:** Zhizhang Wang, Jitao Ji, Xin Ye, Yuxin Chen, Xueyun Li, Wange Song, Bin Fang, Ji Chen, Shining Zhu, Tao Li

**Affiliations:** College of Engineering and Applied Sciences, National Laboratory of Solid State Microstructure, Key Laboratory of Intelligent Optical Sensing and Manipulation, Jiangsu Key Laboratory of Artificial Functional Materials, Nanjing University, Nanjing, 210093, China

**Keywords:** deflection magnification, metasurface-doublet, on-chip integration, optical phased array

## Abstract

Optical phased array (OPA), as a promising beam steering technology, however, usually suffers from a narrow field of view (FOV) that limits its performances in applications. A miniaturized compact strategy to enlarge the beam steering angle is quite desirable for the solid-state OPA technique. Here an on-chip metasurface-doublet is proposed to offer angle magnification integrated with a port-selected optical phased array. It is implemented by combing convex and concave metalenses with the quadratic phase distribution, which is precisely integrated on the OPA chip by layer-by-layer fabrication process. Here, the OPA is fabricated in Lithium Niobate on Insulator (LNOI) platform. Our experiments show that the metasurface-doublet is able to achieve 1.54 times FOV amplification in a horizontal direction and with >41% working efficiency. Our results provide a feasible approach to achieve enlarged FOV for wide-angle beam steering and also imply a powerful platform in developing integrated multilayer metasurface devices.

## 1 Introduction

Light detection and ranging (LiDAR) is important in long-range and high-precision 3D sensing for autonomous vehicles, intelligent perception, and other applications [1], [2], [3], [4]. Optical phased array (OPA) as a promising all-solid-state LiDAR has attracted significant attention due to its high integration, fast speed and compact size [5], [6], [7], [8]. Compared with mechanical LiDAR, the beam steering of OPA technology usually suffers from a narrow field of view (FOV), which limits its further applications [9, 10]. In recently years, metasurface has attracted considerable attention due to its powerful capability in manipulating the amplitude, phase and polarization of light flexibly [11], [12], [13], [14]. Some designs based on metalenses have been implemented to realize a wide FOV such as metalens arrays [15, 16], quadratic phase profile [17, 18], and computational thin-plate lens [19, 20]. More importantly, metasurfaces offer an alternative approach for realization of optical systems in a flat, ultra-thin and lightweight form [21], [22], [23], [24], which promises an effective and compatible scheme to enhance beam steering of all-solid-state OPA devices. For example, it is possible to integrate a single-layer metasurface on OPA to enlarge the FOV by multi-beam steering [25]. However, the deflection angle is not actually enlarged. In principle, it is hard to access a fixed magnification factor by using a single-layer metasurface with a designed phase distribution, since it requires the device adds different in-plane momentum components to beam with various incident angles [26].

Recently, metasurface-doublet have been proposed in research to improve the performance of optical systems and achieve miniaturization of devices [27], [28], [29], such as microscopes [30, 31], spectrometers [32], multifunctional holography [33, 34] and telescopes [35, 36]. Especially, the metasurface-doublet combined with OPAs has been demonstrated to enlarge FOV [37, 38]. However, in those works the expansion of the steering FOV were implemented by combining metasurfaces and solid-state OPAs in the spatial optical setting without showing the advantages the compact solid-state device. In fact, compact integration of layered metasurfaces with other functional optical devices is still challenging.

Here, we propose and experimentally demonstrate an on-chip integration of metasurface-doublet on a port-selected OPA with an enlarged steering range. Through a sophisticated fabrication process, a metasurface-doublet is successfully integrated with the OPA chip on the Lithium Niobate-on-Insulator (LNOI) platform. This on-chip metasurface-doublet consists of convex and concave metalenses with the quadratic phase distribution, which gives rise to 1.54 times FOV expansion in a horizontal dimension and 41% optical efficiency within the steering angle range. Our work shows the capability of the on-chip metasurface-doublet to enlarge FOV for a wide-angle beam steering and promises a feasible scheme for multilayered metasurface integrated devices with enhanced functionalities.

## 2 Principle and design

**Figure 1:**
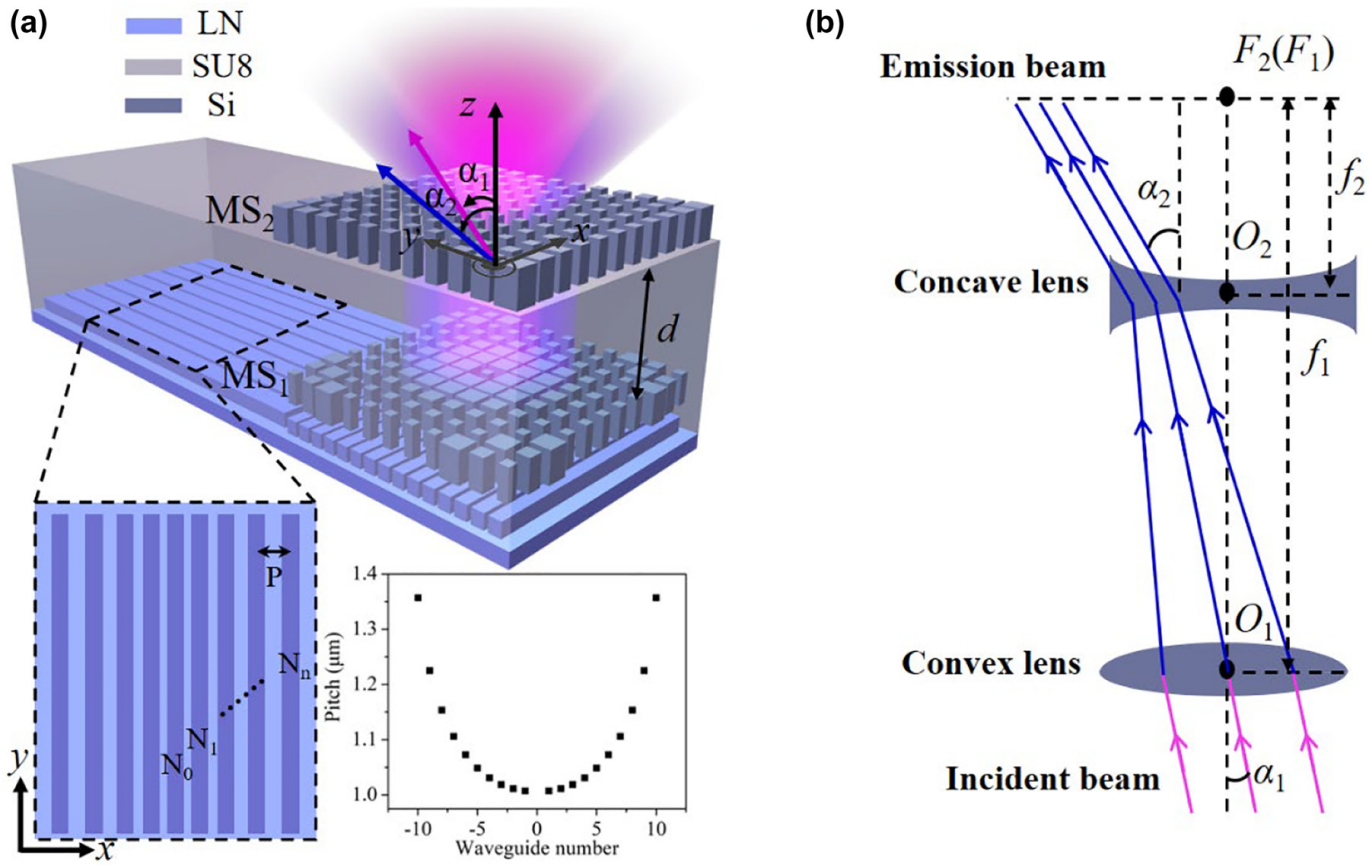
A conceptual schematic of the on-chip metasurface-doublet. (a) Schematic configuration of the proposed on-chip metasurface-doublet integrated with OPA. Inset: the top view of the aperiodic waveguide array and the pitch distributions of waveguides. (b) Conceptual scheme of metasurface-doublet for amplification of beam steering.

Figure 1(a) illustrates the schematics of the proposed on-chip metasurface-doublet integrated with OPA. At the bottom of the device, the OPA is implemented on the LNOI platform and featured with an aperiodic waveguide array, which is designed analogous to the matrix elements of the angular momentum J_x_-operator and serves as an in-plane Fourier Transform to modulate the wavefront of radiation beams to desired deflection angles. The inset in Figure 1(a) exhibits the top view of the aperiodic waveguide array and the corresponding pitch distributions of waveguides. As a proof-of-concept demonstration, the light is imported from multiple input ports into the waveguide array, and then transferred to scattering grating part (underneath the MS_1_) with different angles. More details of the port-selected OPA can be referred to previous work [25]. Although this OPA has demonstrated the capability of free-space beam steering, the FOV is limited and restrict its application performances, such as LiDAR, free-space communications and so on. To enlarge the deflection angles of the radiation beams, here a metasurface-doublet is decorated on top of the OPA, supported by a buffer layer of SU-8 polymer. Similar to the configuration of Galileo telescope system, the metasurface-doublet is made of a convex lens and a concave lens, marked as MS_1_ and MS_2_. Both MS_1_ and MS_2_ are composed of silicon meta-atoms and the gap d between MS_1_ and MS_2_ depends on the magnification factor of the metasurface-doublet. Figure 1(b) describes the principle of the metasurface-doublet that applied for amplification of beam steering in terms of the ray-optical behavior, where the concave lens with focal length of *f*_1_ plays a role as the eyepiece and the convex lens with focal length of *f*_2_ serves as the objective. When an incident beam with oblique angle of *α*_1_ propagates through the convex lens, it is subsequently bent inward and then expanded outward by the concave lens with an increased deflection angle of *α*_1_ = *M* · *α*_2_. Under this condition, the concave lens and the convex lens are confocal, determining the magnification factor to be

(1) $$M=\frac{\alpha_{2}}{\alpha_{1}}\approx -\frac{f_{1}}{f_{2}}.$$

Therefore, an on-chip metasurface-doublet based on Galileo telescope system is implemented to enlarge the deflection angles to acquire large FOV of OPA scanning in a compact integrated form.

**Figure 2:**
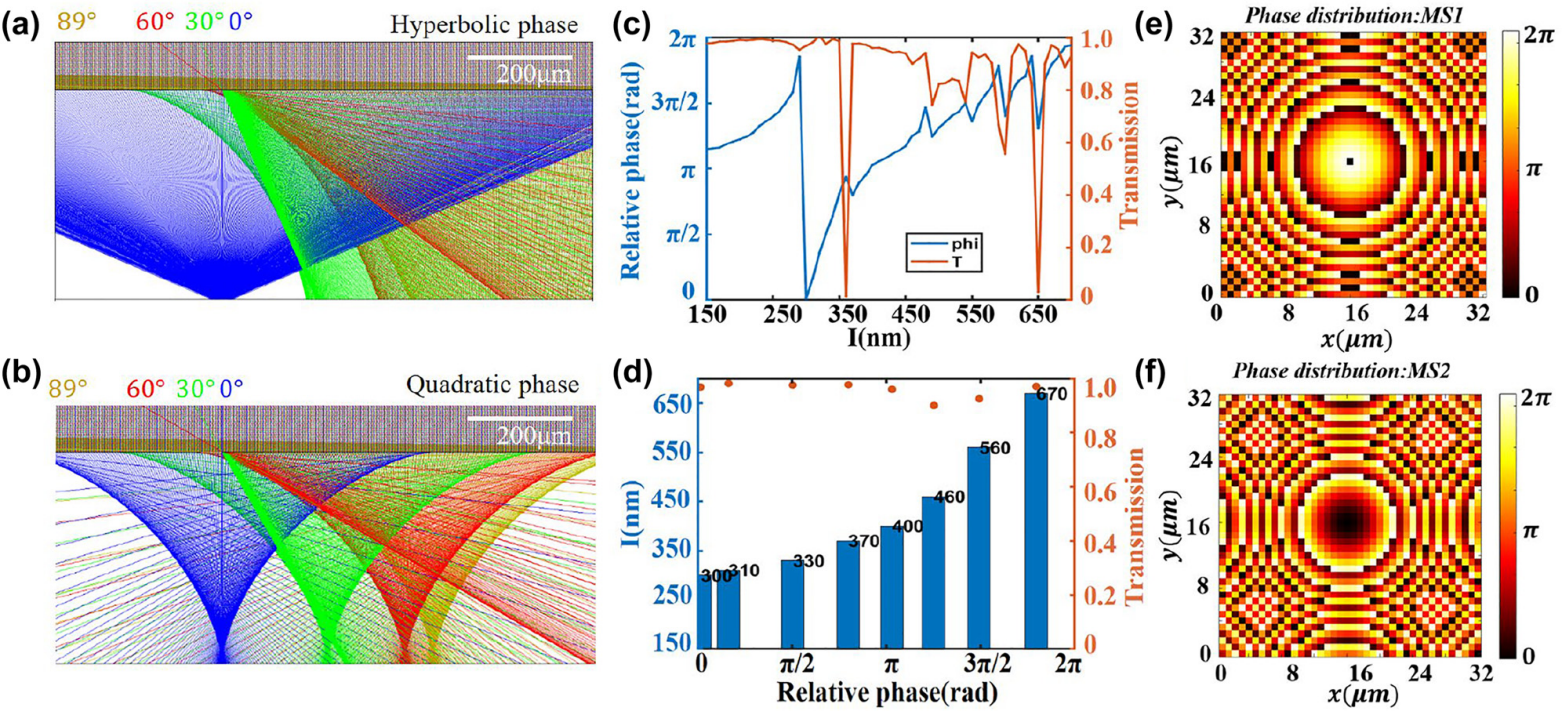
Demonstration of the focusing performances of (a) hyperbolic phase and (b) quadratic phase by Zemax. (c) Simulated phase shift and transmission as a function of the lengths of square nanopillars at 1550 nm. (d) Details of the eight selected square nanopillars. Designed quadratic phase distributions of (e) MS_1_ and (f) MS_2_.

In order to access a wide-angle lensing, quadratic phase type metalens is employed due to its perfect conversion from rotational symmetry to translational symmetry that enables a larger FOV [14, 20]. For comparison, four beams with different incident angles (0°, 30°, 60° and 89°) impinging upon the interfaces with hyperbolic phase and quadratic phase, respectively, are simulated by Zemax to investigate their focusing performances. As illustrated in Figure 2(a) and (b), the hyperbolic phase can hardly focus the light with incident angle larger than 30°, while the quadratic phase is of significant capability to focus light even at the large incident angle to 89°. Therefore, in our design, quadratic phase is adopted to the designs of MS_1_ and MS_2_ to achieve wide-angle beam steering. The distributions of quadratic phase can be expressed as

(2) $$\phi_{\text{quad}}\left(r\right)=\frac{-k_{0}r^{2}}{2f}n_{\text{ext}},$$

where *f*, *k*_0_ and *n*_ext_ are the focal length, wave number and the refractive index of surrounding medium, respectively.

To realize the on-chip integration, the metasurface-doublet including MS_1_ and MS_2_ is constituted of silicon square nanopillars with a height of 1 μm and arranged in a lattice constant of 800 nm. As illustrated in Figure 2(c), the simulated phase shift covers a span of 0 ∼ 2*π* by regulating the lengths of square nanopillars at operating wavelength of 1550 nm. Eight square nanopillars with lengths ranging from 300 to 670 nm with transmission over 90% (see Figure 2(d)) are selected for the subsequent design of concave and convex metalenses to guarantee the high efficiency of the metasurface-doublet. The doublet metalenses (MS_1_ and MS_2_) are designed with focal lengths of *f*_1_ and *f*_2_ (negative value) and arranged with a distance of *d* = *f*_1_ + *f*_2_, rendering a magnification factor *M* = |*f*_1_/*f*_2_|. According to Equation (2), the quadratic phase profiles of MS_1_ and MS_2_ are derived and presented in Figure 2(e) and (f), respectively. With the on-chip integration of the metasurface-doublet and OPA, the beam radiating from the grating antennas of the OPA is modulated without any post-fabrication alignment. Hence, the deflection angles of the radiation beams can be ultimately amplified by a factor of *M* in a chiplet manner, which features an ultra-compact footprint compared with its free-space counterpart.

## 3 Results

**Figure 3:**
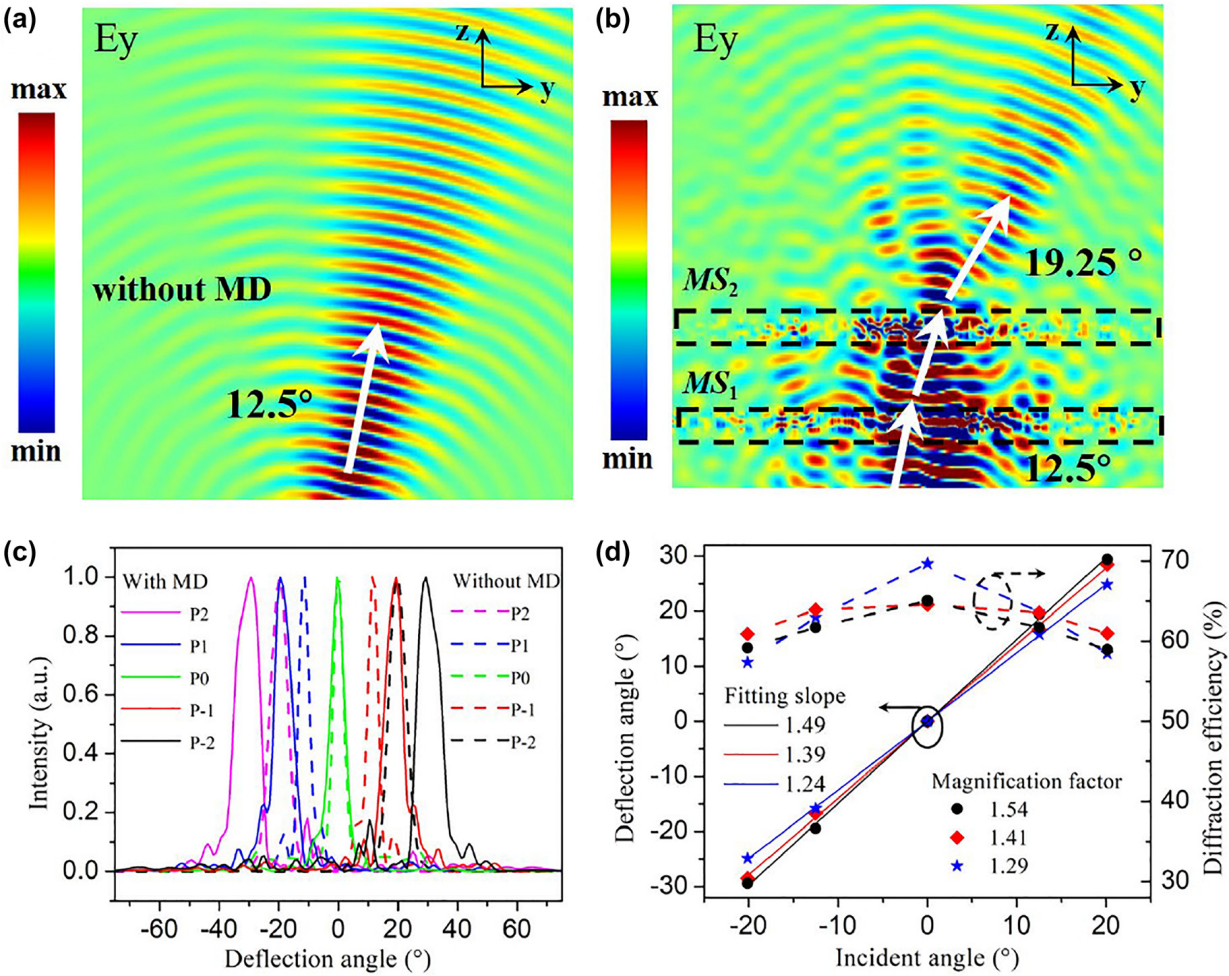
Simulated electric field distributions under 12.5° oblique incidence (a) without and (b) with metasurface-doublet at 1550 nm. (c) Simulated deflection angles when switching five input ports of the port-selected OPA with/without metasurface-doublet. (d) Deflection angles and diffraction efficiencies as a function of incident angles under three magnification factors.

To investigate the feasibility of the proposed metasurface-doublet, numerical simulations are performed via three-dimensional finite-difference time-domain (FDTD) method. The simulation model is based on the configuration as illustrated in Figure 1(a). The fundamental TE mode sources of 1550 nm are applied to input ports for beam steering with desired angles. The slab grating antennas at the end of the aperiodic waveguide array are designed to emit the guided wave into space. With a buffer layer of SU-8, the metasurface-doublet with particularly designed phase distributions is positioned on top of the grating antennas. Perfect matched layer (PML) is set as boundary condition along all the directions in the simulation. Figure 3(a) and (b) show the electric field distributions under 12.5° oblique incidence without and with metasurface-doublet of a magnification factor *M* of 1.54. It shows that the incident beam with deflection angle of 12.5° is enlarged to 19.25° after the metasurface-doublet, corresponding to the magnification factor of 1.54. Figure 3(c) presents the simulated deflection angles when switching five input ports of the OPA with and without metasurface-doublet from port P-2 to P2. The dashed lines represent the deflection angles of the base OPA without metasurface-doublet, exhibiting the whole FOV ranging from −20.14 to 20.14°. By integrating the OPA with metasurface-doublet of *f*_1_ = 19.3 μm and *f*_2_ *=* −12.5 μm, the deflection angles are significantly expanded to ±29.4°, see the solid lines in Figure 3(c). To further evaluate the magnification capability of the metasurface-doublet, the deflection angles as a function of the incident angles are plotted in Figure 3(d) according to three designed magnification factors of 1.29, 1.41 and 1.54, respectively. Through linear fitting of the deflection angles, the simulated magnification factors are revealed by the slopes of the three lines in Figure 3(d) to be 1.24, 1.39 and 1.49. The simulated magnification factors match well with the designed ones, demonstrating the feasibility of the metasurface-doublet. In addition, the diffraction efficiency, defined as the ratio of the deflected optical power to the input optical power flowing into metasurface-doublet, reaches up to 70% and tends to decrease with the enlarged incident angle. Nevertheless, the diffraction efficiencies of all the devices remain above 58%, thanks to the high transmission of the meta-atoms and the elaborate design of quadratic metalens.

**Figure 4:**
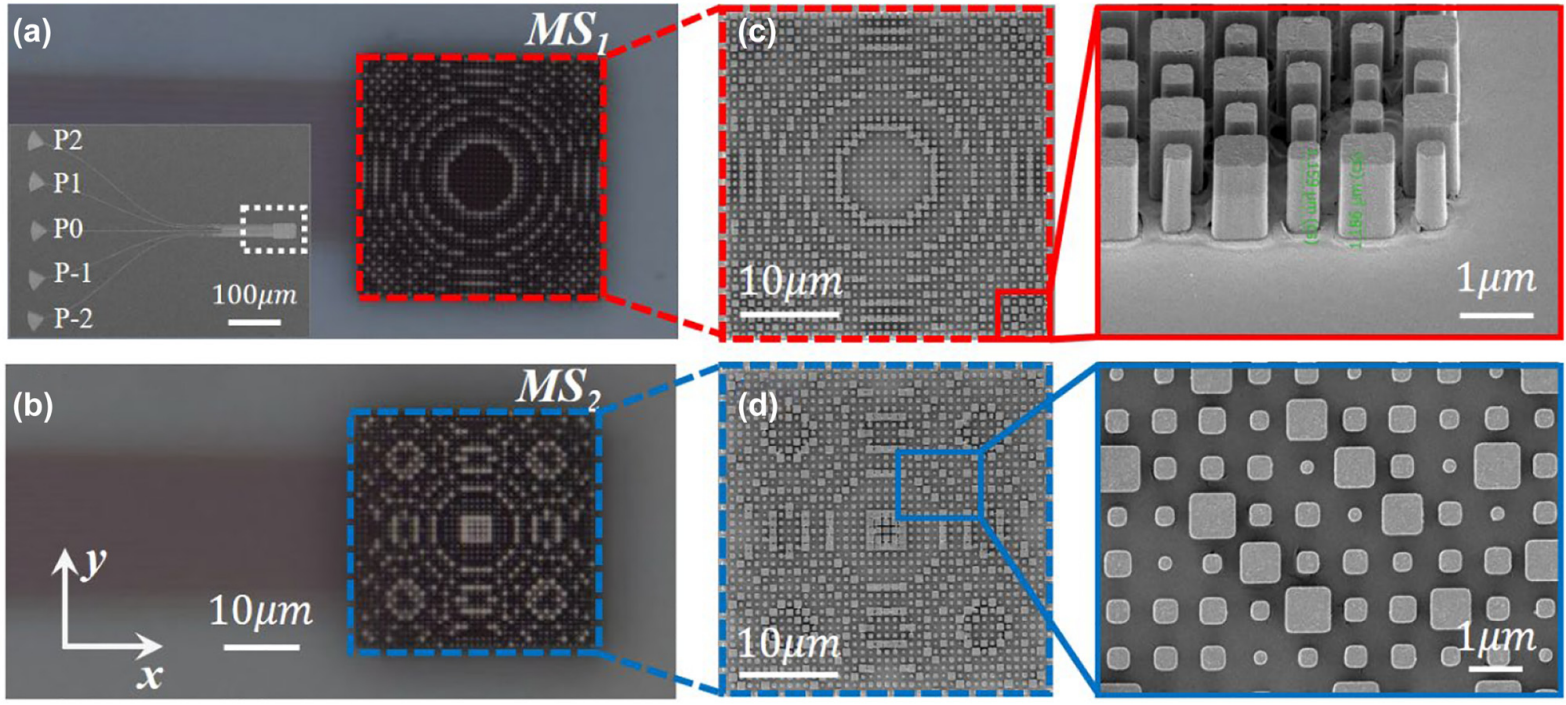
Optical microscopy images of the fabricated (a) MS_1_ and (b) MS_2_ mounted on top of the OPA. Inset: top view of the OPA with five input ports. The white dashed line indicates the location of MS_1_ and MS_2_. Scanning electron microscopy (SEM) images of (c) MS_1_ and (d) MS_2_. Left side: overall perspective of MS_1_ and MS_2_. Right side: side and top view of the details indicated by red and blue solid lines.

After the numerical investigation of the performance of the device, the metasurface-doublet is prepared on top of the OPA implemented in LNOI platform. First, a SU-8 layer was spin-coated to fill the waveguide gaps and cover the grating antenna of the OPA, acting as a buffer layer to protect the structure of OPA. Then, MS_1_ composed of amorphous Si nanopillars were decorated on the SU-8 through subsequent plasma-enhanced chemical vapor deposition (PECVD), E-beam lithography (EBL) and dry etching process. To protect the fabricated MS_1_ from damage, another SU-8 layer was employed to cover MS_1_ and provide a flat substrate for MS_2_ with a distance *d* from MS_1_. As a last step, MS_2_ was defined on top of SU-8 buffer layer through the same process of MS_1_. The optical microscopy images of the fabricated MS_1_ and MS_2_ mounted on top of the OPA are illustrated in Figure 4(a) and (b), respectively. The insets in Figure 4(a) shows the top view of the OPA with five input ports, where the location of the metasurface-doublet is highlighted by the white dashed line. Figure 4(c) and (d) exhibit the SEM images of the details of MS_1_ and MS_2_, respectively. It should be noted that the focal length of MS_1_ is fixed to be *f*_1_ = 19.3 μm and the focal length of MS_2_ can be variable to achieve different magnification factors. For comparison, three different focal lengths of MS_2_, −15 μm, −13.7 μm and −12.5 μm, were also fabricated to enlarge the deflection angles by a factor of *M* = 1.29, 1.41 and 1.54.

**Figure 5:**
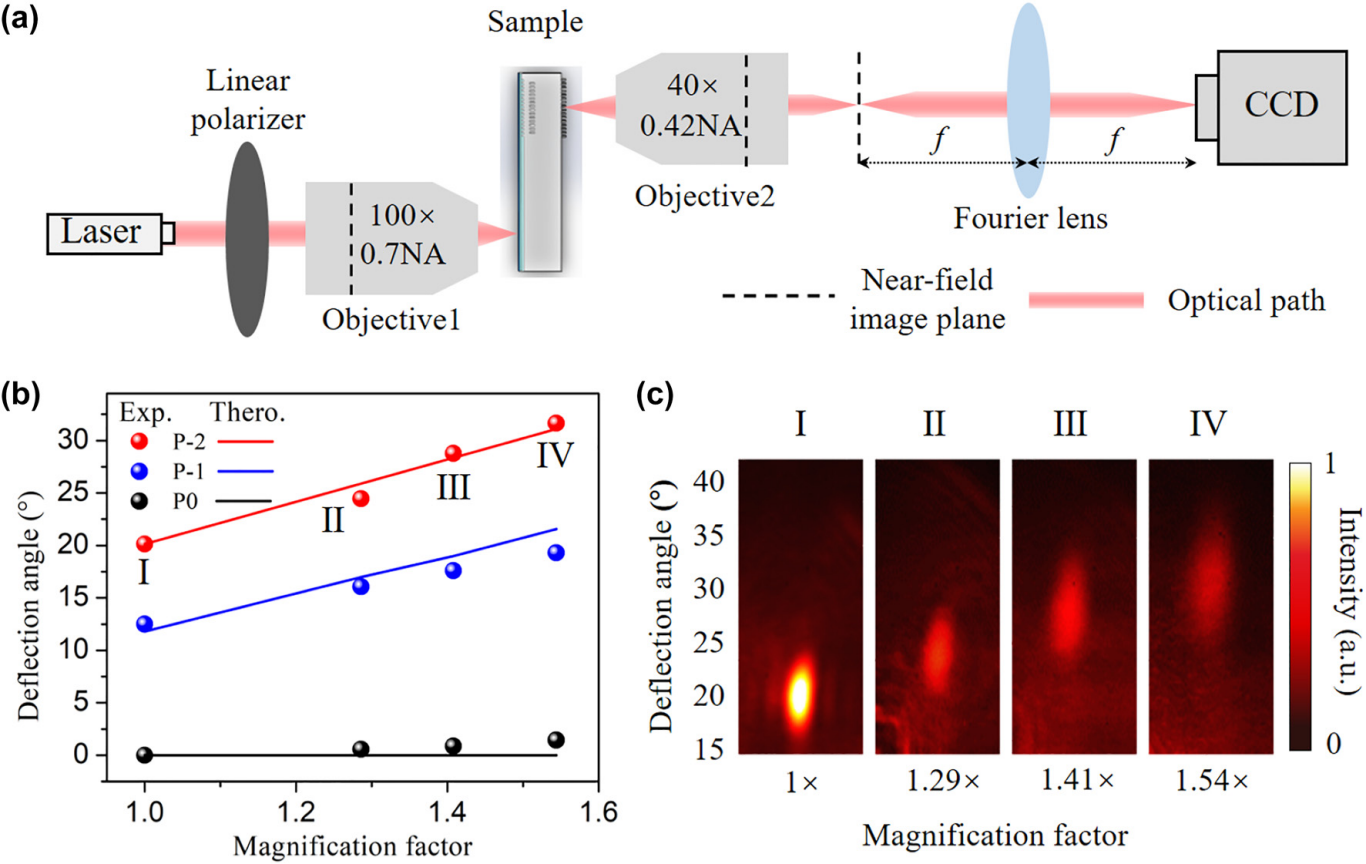
Experimental results of the angle magnification by metasurface-doublet. (a) A schematic of the experimental setup for far-field optical measurements. (b) Deflection angles of the metasurface-doublet with different magnification factors when illuminating input port P-2 to P0. (c) Measured far-field intensity distributions under different magnification factors when illuminating input port P-2.

To experimentally characterize the performance of the on-chip metasurface-doublet, an experimental setup is established as schematically shown in Figure 5(a). A 1550 nm-wavelength ps-pulsed laser filtered from a white light laser (Fianium Super-continuum, 4W) followed with a linear polarizer is focused onto the put grating couplers through an objective with numerical aperture (NA) of 0.70. Then the guided wave is transferred via the aperiodic waveguide array to the output grating antenna for emission. The light emitted through the metasurface-doublet is collected by another objective with a NA of 0.42. Subsequently, a lens is utilized to perform Fourier transformation of the object plane into a near-infrared CCD (XenicsXeva-1.7–320).

On the basis of OPA, the deflection angles of the metasurface-doublet with three different *f*_2_ which represent three magnification factors are characterized by switching the input ports from P0 to P-2. As demonstrated in Figure 5(b), the deflection angles of the metasurface-doublet are amplified from 20.14 to 24.34°, 28.82 and 31.25° when the input port P-2 is feed, according to the magnification factor of 1.29, 1.41 and 1.54 respectively, where magnification factor of 1 represents that of bare OPA. This experimental results coincide with the theoretical predicts indicated by the red solid line, confirming the capability of the metasurface-doublet for enlargement of beam scanning. Additionally, when switching the input port P-1 and P0, the deflections angles of the metasurface-doublet are close to the theoretical values indicated by blue and black lines with negligible deviations. Corresponding to the four conditions marked by Roman numerals in Figure 5(b) and (c) displays the measured far-field intensity distributions of three metasurface-doublets with different magnification factors when the input port P-2 is fed. The diffraction efficiencies of these three metasurface-doublets under 20.14° incidence are characterized to be 70%, 55% and 41%, respectively. It is worth mentioning that the size of the incident beam is also expanded both along the transverse and longitudinal directions under the modulation of the metasurface-doublet. The beam inevitably divergences by a factor of *M* as the deflection angles are enlarged at the cost of the angular resolution. The problem of angular resolution deterioration may be mitigated by expanding the radiation aperture of the OPA and specific design of the metasurface-doublet (e.g., adding an additional focusing phase with respect to a certain near-field working distance).

## 4 Discussion and conclusion

By integrating metasurface-doublet with OPA on the same chip, the function of enhanced beam deflection is realized, providing a feasible compact scheme for the realization of wide-angle optical scanning system. Although the magnification factor of the metasurface-doublet achieved in this work is not very large, it can work under the condition of large incident angles due to the quadratic phase designs. This feature is distinguished from previously proposed metasurface-doublet with relative larger magnification factors but only working at ultra-small incident angles [37, 38]. Meanwhile, the magnification factor of this metasurface-doublet can be further improved by optimizing the focal lengths of MS_1_ and MS_2_ according to the required FOV and angular accuracy. To verify the amplification function of the metasurface-doublet, the deflection angles ranging from 0 to 30° are demonstrated in this work and a larger deflection angle is also accessible by adjusting the design of OPA for an increased incident angle. In addition, it should be emphasized that the metasurface-doublet can be compatible with other on-chip beam scanning systems to acquire on-chip dynamic wide-angle beam steering. By virtue of the SU-8 buffer layer, a fabrication method of on-chip integration of metsurface doublet has been proposed. Hence, there is no need of any other spatial alignment, showing the advantages of integration and miniaturization over its free-space counterparts.

In summary, a metasurface-doublet has been successfully integrated with OPA implemented on LNOI platform, achieving angular magnification for on-chip beam steering. The on-chip metasurface-doublet composed of a concave lens and a convex lens, has demonstrated its magnification capability for wide-angle beam steering with high efficiency based on quadratic metalens design. Through switching the five input ports of the OPA integrated with metasurface-doublet, the largest deflection angles of the radiated beams can be enlarged from 20.9 to 31.25°. Moreover, the magnification factors of the metasurface-doublet of 1.29, 1.41 and 1.54 have been achieved experimentally, which are fully controllable by adjusting the focal lengths and the distance between MS_1_ and MS_2_. Benefiting from the precise and sophisticated fabrication process, the prominent advantage of the metasurface-doublet lies on its on-chip integration compatibility, eliminating the requirement of complex alignment processes which are inevitable for free-space metasurface-doublet systems. The proposed on-chip metasurface-doublet provides a feasible approach to achieve enlarged FOV for wide-angle beam steering and indicates a promising direction for the integration of metasurface and integrated photonics devices.

## References

[1] Hsu C. P., Li B., Solano-Rivas B. (2021). A review and perspective on optical phased array for automotive LiDAR. IEEE J. Sel. Top. Quantum Electron..

[2] Zhang X., Kwon K., Henriksson J., Luo J., Wu M. (2022). A large-scale microelectromechanical-systems-based silicon photonics LiDAR. Nature.

[3] Fukui T., Kohno Y., Tang R., Nakano Y., Tanemura T. (2021). Single-pixel imaging using multimode fiber and silicon photonic phased array. J. Lightwave Technol..

[4] Kim I., Martins R. J., Jang J. (2021). Nanophotonics for light detection and ranging technology. Nat. Nanotechnol..

[5] Heck M. J. R. (2017). Highly integrated optical phased arrays: photonic integrated circuits for optical beam shaping and beam steering. Nanophotonics.

[6] Guo Y., Guo Y., Li C., Zhang H., Zhou X., Zhang L. (2021). Integrated optical phased arrays for beam forming and steering. Appl. Sci..

[7] Li C., Cao X., Wu K. (2021). Blind zone-suppressed hybrid beam steering for solid-state Lidar. Photon. Res..

[8] Gozzard D. R., Roberts L. E., Spollard J. T., Sibley P. G., Shaddock D. A. (2020). Fast beam steering with an optical phased array. Opt. Lett..

[9] Li Y., Chen B., Na Q. (2021). Wide-steering-angle high-resolution optical phased array. Photon. Res..

[10] Liu Y., Hu H. (2022). Silicon optical phased array with a 180-degree field of view for 2D optical beam steering. Optica.

[11] Yu N., Capasso F. (2014). Flat optics with designer metasurfaces. Nat. Mater..

[12] Kildishev A. V., Boltasseva A., Shalaev V. M. (2013). Planar photonics with metasurfaces. Science.

[13] Arbabi A., Horie Y., Bagheri M., Faraon A. (2015). Dielectric metasurfaces for complete control of phase and polarization with subwavelength spatial esolution and high transmission. Nat. Nanotechnol..

[14] Luo X. G., Zhang F., Pu M. B. (2022). Recent advances of wide-angle metalenses: principle, design, and applications. Nanophotonics.

[15] Chen J., Ye X., Gao S. (2022). Planar wide-angle-imaging camera enabled by metalens array. Optica.

[16] Ye X., Qian X., Chen Y. (2022). Chip-scale metalens microscope for wide-field and depth-of-field imaging. Adv. Photon..

[17] Peng Y., Sun Q., Dun X. (2019). Learned large field-of-view imaging with thin-plate optics. ACM Trans. Graph..

[18] Hao C., Gao S., Ruan Q. (2020). Single-layer aberration-compensated flat lens for robust wide-angle imaging. Laser Photon. Rev..

[19] Pu M., Li X., Guo Y., Ma X., Luo X. (2017). Nanoapertures with ordered rotations: symmetry transformation and wide-angle flat lensing. Opt. Express.

[20] Martins A., Li K., Li J. (2020). On metalenses with arbitrarily wide field of view. ACS Photonics.

[21] Reshef O., DelMastro M. P., Bearne K. K. M. (2021). An optic to replace space and its application towards ultra-thin imaging systems. Nat. Commun..

[22] Xu B. B., Li H. M., Gao S. L. (2020). Metalens-integrated compact imaging devices for wide-field microscopy. Adv. Photon..

[23] Fang B., Wang Z., Gao S., Zhu S., Li T. (2022). Manipulating guided wave radiation with integrated geometric metasurface. Nanophotonics.

[24] Meng Y., Chen Y., Lu L. . (2021). Optical meta-waveguides for integrated photonics and beyond. Light Sci. Appl..

[25] Wang Z., Song W., Chen Y. (2022). Metasurface empowered lithium niobate optical phase array with enlarged field-of-view. Photon. Res..

[26] He Z., Yin K., Wu S. T. (2021). Miniature planar telescopes for efficient, wide-angle, high-precision beam steering. Light Sci. Appl..

[27] Arbabi A., Arbabi E., Kamali S. M., Horie Y., Han S., Faraon A. (2016). Miniature optical planar camera based on a wide-angle metasurface doublet corrected for monochromatic aberrations. Nat. Commun..

[28] Groever B., Chen W. T., Capasso F. (2017). Meta-lens doublet in the visible region. Nano Lett..

[29] Arbabi A., Arbabi E., Kamali S. M. (2016). Miniature optical planar camera based on a wide-angle metasurface doublet corrected for monochromatic aberrations. Nat. Commun..

[30] Faraji-Dana M. S., Arbabi E., Arbabi A. (2018). Compact folded metasurface spectrometer. Nat. Commun..

[31] Arbabi E., Arbabi A., Kamali S. M. (2018). MEMS-tunable dielectric metasurface lens. Nat. Commun..

[32] Kwon H., Arbabi E., Kamali S. M. (2020). Single-shot quantitative phase gradient microscopy using a system of multifunctional metasurfaces. Nat. Photon..

[33] Zhou Y., Kravchenko I., Wang H. (2019). Multifunctional metaoptics based on bilayer metasurfaces. Light Sci. Appl..

[34] Deng L., Li Z., Zhou Z. (2022). Bilayer-metasurface design, fabrication, and functionalization for full-space light manipulation. Adv. Opt. Mater..

[35] Liu X., Deng J., Li K. F. (2020). Optical telescope with Cassegrain metasurfaces. Nanophotonics.

[36] Zhou C., Lee B W. B., Park C. S. (2020). Multifunctional beam manipulation at telecommunication wavelengths enabled by an all-dielectric metasurface doublet. Adv. Opt. Mater..

[37] Lee W. B., Im C. S., Zhou C. (2022). Metasurface doublet-integrated bidirectional grating antenna enabling enhanced wavelength-tuned beam steering. Photon. Res..

[38] Li H., Zhou C., Lee W. B. (2022). Flat telescope based on an all-dielectric metasurface doublet enabling polarization-controllable enhanced beam steering. Nanophotonics.

